# A self-destructive nanosweeper that captures and clears amyloid β-peptides

**DOI:** 10.1038/s41467-018-04255-z

**Published:** 2018-05-04

**Authors:** Qiang Luo, Yao-Xin Lin, Pei-Pei Yang, Yi Wang, Guo-Bin Qi, Zeng-Ying Qiao, Bing-Nan Li, Kuo Zhang, Jing-Ping Zhang, Lei Wang, Hao Wang

**Affiliations:** 1 Faculty of Chemistry, Northeast Normal University, 130024 Changchun, China; 2 CAS Center for Excellence in Nanoscience, CAS Key Laboratory for Biomedical Effects of Nanomaterials and Nanosafety, National Center for Nanoscience and Technology (NCNST), 100190 Beijing, China; 3 University of Chinese Academy of Sciences, 100049 Beijing, China; 4 School of Pharmaceutical Sciences (Shenzhen), Sun Yat-sen University, Guangzhou, 510006 China

## Abstract

Cerebral amyloid β-peptide (Aβ) accumulation resulting from an imbalance between Aβ production and clearance is one of the most important causes in the formation of Alzheimer’s disease (AD). In order to preserve the maintenance of Aβ homeostasis and have a notable AD therapy, achieving a method to clear up Aβ plaques becomes an emerging task. Herein, we describe a self-destructive nanosweeper based on multifunctional peptide-polymers that is capable of capturing and clearing Aβ for the effective treatment of AD. The nanosweeper recognize and bind Aβ via co-assembly through hydrogen bonding interactions. The Aβ-loaded nanosweeper enters cells and upregulates autophagy thus promoting the degradation of Aβ. As a result, the nanosweeper decreases the cytotoxicity of Aβ and rescues memory deficits of AD transgenic mice. We believe that this resourceful and synergistic approach has valuable potential as an AD treatment strategy.

## Abstract (web summary)

Cerebral amyloid β-peptide accumulation is a causative factor in Alzheimer’s Disease. Here the authors design a 'nanosweeper' that binds amyloid β-peptide and induces autophagy to clear the accumulated plagues.

## Introduction

Alzheimer’s disease (AD) has been considered the most pervasive neurodegenerative disorder, affecting a great number of humans worldwide^1–3^. The emphatic pathological hallmarks of AD are extracellular deposits of self-assembled fibrils based on the amyloid β-peptide (Aβ) and intracellular neurofibrillary tangles containing hyper-phosphorylated tau^4–8^. Over the past few decades, the trend in therapeutic methods for AD involved inhibiting the self-assembly of Aβ into fibrils and thus the resultant deposition of Aβ. Intense research indicates that inhibitors, such as antibodies^9^, peptide-based nanomaterials^10–12^, small molecules^13,14^, and various nanoparticles^15–17^, can decelerate the aggregation of Aβ. However, the recent failure of clinical trials based on bapineuzumab^18^ and solanezumab^19^ suggests that there is still a long way to go in treatment of AD against Aβ^20,21^. It has been accepted, when considering AD therapeutics, that the clearance of Aβ is the essential component in the maintenance of Aβ homeostasis.

Autophagy, the way by which cells degrade their own metabolites, may be applied for Aβ clearance. Indeed, the dysfunction of the autophagy-lysosome system leads to the accumulation of Aβ^22,23^. It is beneficial for the enhancement of Aβ clearance that the induction of autophagy is appropriately elevated, giving rise to the distinct implication of autophagy as a therapeutic strategy^23–25^. However, because Aβ generated from amyloid precursor protein (APP) in endosomes is recycled to the cell surfaces and usually aggregates and deposits outside the cell while autophagic degradation occurs intracellularly, the concept of Aβ clearing by autophagy is contradictory.

**Fig. 1:**
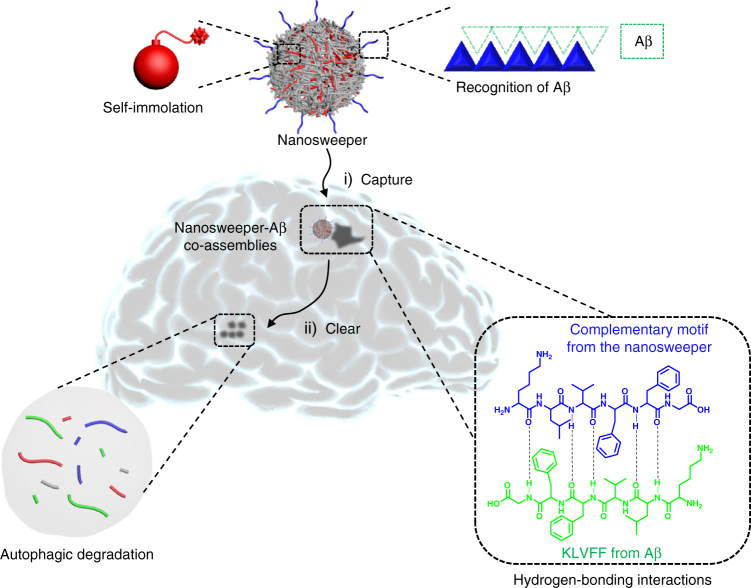
The schematic illustration of the nanosweeper mechanism of action. The nanosweeper captures Aβ by hydrogen-bonded co-assembly and internalizes a substantial amount into cells carrying Aβ. Then, the nanosweeper activates the cell’s autophagic response, resulting in the degradation of Aβ and the nanosweeper itself

Herein, we design a nanosweeper with the goal of effectively degrading extracellular Aβ, and find that it not only captures extracellular Aβ and carries Aβ into cells, but it also up-regulates cellular autophagy and digestes Aβ. The nanosweeper is composed of a cationic chitosan (CS) core decorated with PEGylated-GKLVFF (designated as K) and Beclin-1 (TGFQGSHWIHFTANFVNT, designated as B). KLVFF can recognize and co-assemble with Aβ through hydrogen-bonding interactions. Beclin-1 can induce autophagy to degrade Aβ. The polyethylene glycol (PEG) increases the dispersity of the nanosweeper in water, providing the appropriate biocompatibility and stability. First, the nanosweeper captures and co-assembles with extracellular Aβ specifically, inhibiting the formation of toxic Aβ aggregates remarkably. Next, the nanosweeper preferentially delivers Aβ into cells and activates autophagy within them to degrade Aβ, ultimately resulting in Aβ clearance (Fig. 1). Results from in vitro and in vivo experiments confirms the nanosweeper’s high-efficiency for Aβ clearance. Among the Aβ-treated cells, the nanosweeper increases the cell viability from approximately 60 to 93%. Furthermore, the insoluble Aβ is decreased from 1539 to 914 ng/mg, and soluble Aβ is decreased from 585 to 190 ng/mg in the brain of AD transgenic mice treated with the nanosweeper, leading to rescued memory deficits. This delicate nano-strategy can be a potential therapeutic approach in the treatment of AD.

## Results

### Preparation of the nanosweeper

**Fig. 2:**
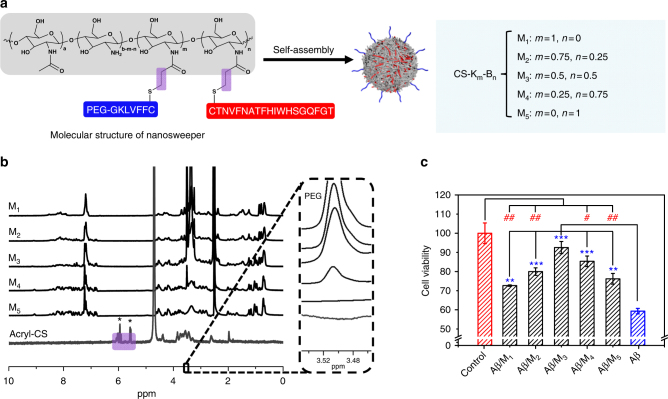
The molecular structure and biological effect of M_3_ in vitro. **a** Molecular structures of the multifunctional peptide-polymer M_1–5_, K and B were the abbreviation of HS-CFFVLKG-PEG (capture unit) and HS-CTNVFNATFHIWHSGQFGT (clearance unit), respectively. **b** The ^1^H NMR of M_1–5_ in ^6^D-DMSO and acryl-CS in ^6^D-H_2_O. **c** Aβ cytotoxicity (20 μM) to N2a cells was reduced in the presence of 20 μg·mL^−1^ M_1–5_. Data are presented as mean ± standard deviation (s.d.) (*n* = 3), analyzed by a Student’s *t*-test. Statistical significance is indicated as *p < 0.05, **p < 0.01, and ***p < 0.001, for comparison with Aβ group, ^#^*p* < 0.05, ^##^*p* < 0.01, and ^###^*p* < 0.001 for comparison with control group

A series of nanosweepers with various chemical compositions were established by changing the peptide ratios^26^. There were two functional peptide analogs, a KLVFF peptide that could recognize and bind with Aβ_42_^27,28^, and a Beclin1 peptide that could significantly induce autophagy^29,30^. Both functional peptides were applied for linking with chitosan (CS) that was first modified by acrylate (acryl-CS). To control the hydrophobic/hydrophilic balance of the nanosweeper, PEG_368_ was used to modify the G residue of the GKLVFF peptide. As shown in Fig. 2a, the feed molar ratios of KLVFF (Supplementary Fig. 1) and Beclin-1 were adjusted to obtain polymers with various peptide ratios. The final products were designated as M_1_, M_2_, M_3_, M_4_, and M_5_. The structures of acryl-CS and M_1–5_ were confirmed by ^1^H NMR. As shown in Fig. 2b, the ^1^H NMR spectrum of acryl-CS revealed acrylate double bonds at 5.8–6.3 ppm. However, the acrylate double bonds in the ^1^H NMR of M_1–5_ disappeared entirely, indicating that the acrylate groups in acryl-CS reacted completely with sulfhydrylated KLVFF and Beclin-1 peptide. The peaks at 6.7–8.5 in the spectra of M_2_, M_3_, M_4_ and M_5_ were typical Beclin-1 peaks^30^. Meanwhile, a characteristic PEG peak appeared at 3.4–3.6 in the ^1^H NMR spectra of M_1_, M_2_, M_3_ and M_4_, but not in that of M_5_.

### Biocompatibility and anti-Aβ toxicity of the nanosweeper

In order to investigate the biological effects of the nanosweeper, we first explored its biocompatibility by measuring cytotoxicity in the mouse neuroblastoma cell line N2a. Cells were cultured with M_1–5_ for 24 h, and then assessed by CCK-8 assay. As shown in Supplementary Fig. 2, M_1–5_ at a concentration of 20 μg·mL^−1^ showed good biocompatibility with approximate 100% viability, and so this concentration was utilized for further in vitro experiments. Next, to measure the anti-Aβ toxicity of M_1–5_, we detected the cell viability of N2a cells treated with Aβ_42_ (20 μM) in the absence or presence of M_1–5_ (20 μg·mL^−1^). As shown in Fig. 1c, Aβ_42_ alone exhibited obvious toxicity (cell viability, 59.3%), which confimred the findings of previous reports^6,31,32^. However, adding nanosweepers reduced the toxicity of Aβ_42_. M_2–4_ containing both K and B treated groups showed remarkably higher cell viabilities (*p < 0.05, **p < 0.01, ***p < 0.001, Student’s t-test) than M_1_ and M_5_ treated groups, which only contains either K or B. This difference in cell viabilities indicated that the combination of both functional peptides resulted in a synergistic anti-Aβ*-*toxicity effect. M_3_ showed the highest cell viability (92.5%), close to that of the control group (100%) and was screened as the potential lead nanosweeper for clearance of Aβ and AD treatment.

To further verify the synergistic anti-Aβ toxicity effect of M_3_, two control peptide-polymers were prepared, M_K_, in which the Beclin-1 peptide was replaced by a control peptide without autophagy activation functionality, and M_B_, in which the KLVFF peptide was replaced by a KAAGG peptide without Aβ capture functionality (Supplementary Fig. 3). The chemical structures of both control peptide-polymers were also confirmed by ^1^H NMR spectra (Supplementary Fig. 4). The cell viability results of M_K_ (78.0%) and M_B_ (80.9%) implied that they had lower anti-Aβ toxicity than M_3_ (Supplementary Fig. 5), which indicated that the enhanced anti-Aβ-toxicity seen with M_3_ originated from the synergistic effect of KLVFF and Beclin-1.

### Capture of Aβ_42_ by the nanosweeper

**Fig. 3:**
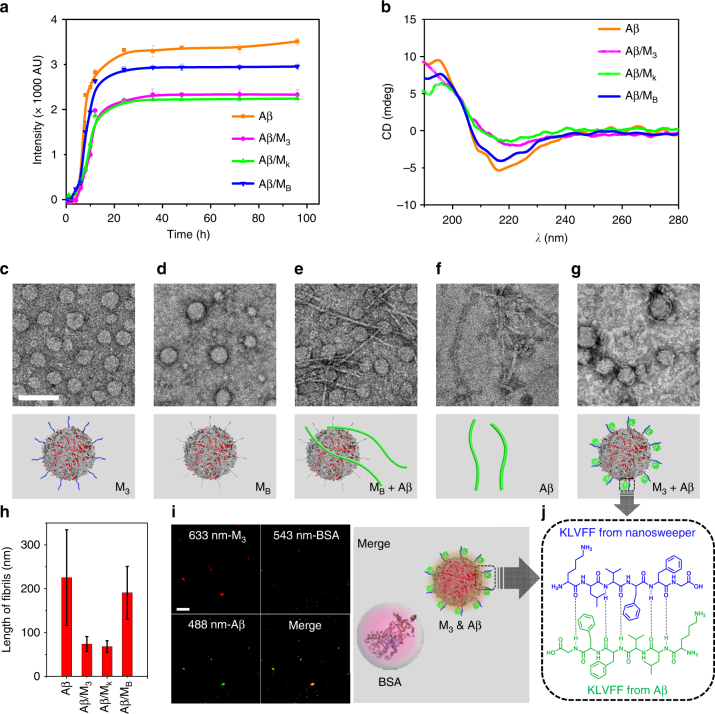
The capture of Aβ by M_3_ through co-assembly. **a** ThT fluorescence assay of Aβ and Aβ/M_3_ in 96 h, with Aβ/M_K_ and Aβ/M_B_ as controls. Data are presented as mean ± s.d. (*n* = 3). **b** The CD spectra of Aβ and Aβ/M_3_ at 24 h, with Aβ/M_K_ and Aβ/M_B_ as controls. **c**–**g** TEM images of M_3_, M_B_, Aβ/M_B_, Aβ and Aβ/M_3_ at 24 h. **h** The statistical length of Aβ fibers was averaged from 5 individual fibers per group. Data are presented as mean ± s.d. (*n* = 5). **i** CLSM images and schematic illustration of the mixture of Cy5-labeled M_3_, FITC-labeled Aβ and Cy3.5-labeled BSA. **j** The schematic illustration of the driving force for the capture of Aβ by hydrogen bonds. Scale bar: white bar in (**c**) is 100 nm, and (**i**) is 20 µm

In order to study the ability of the nanosweeper to regognize and co-assemble with Aβ_42_, we first employed the Thioflavin T (ThT) fluorescence assay to detect Aβ fibril formation in the presence of M_3_. The ThT assay, an established method of dye binding, was applied to monitor Aβ_42_ aggregation because its fluorescent spectrum can be altered with the growth of fibrils^6^. Figure 3a displays a series of sigmoidal curves which are typical of Aβ_42_ fibrillation. The fluorescence intensity of neat Aβ_42_ was higher due to the larger amount of Aβ_42_ fibrillation formation than that of Aβ_42_ in the presence of M_3_, which was similar to the result observed with M_K_, and much lower than that observed with M_B._ The decreasing ThT fluorescence seen from M_5_ to M_1,_ together with the increasing KLVFF content (Supplementary Fig. 6), validated the recognition and co-assembly functionality of KLVFF, resulting in the inhibition of Aβ_42_ aggregation. Furthermore, the results of circular dichromism (CD) spectra confirmed that M_3_ inhibited the formation of β-sheet structrured Aβ_42_ fibrils (Fig. 3b). The Aβ_42_ showed random structures at 0 h (Supplementary Fig. 7) and typical β-sheet structure at 24 h, with positive and negative signals at 195 and 216 nm, respectively. The M_3_ or M_K_ treated Aβ did not show typical β-sheet structures at 24 h, however, the M_B_ treated Aβ_42_ did show typical β-sheet structures at 24 h. The CD measurements also revealed the inhibitory effect of Aβ_42_ fibrillation due to KLVFF recognition and co-assembly.

To provide evidence for supporting the binding effect of M_3_ to Aβ_42_, transmission electron microscopy (TEM) and dynamic light scattering (DLS) were exploited to detect the evolution of morphology and size distribution in an Aβ_42_ solution in the presence of M_3_, with M_B_ and M_K_ acting as control groups. The M_3_, M_B,_ and M_K_ in solution (H_2_O:DMSO = 98:2, 20 μg·mL^−1^) showed similar particulate structures with 43.8 ± 11.1 nm diameter at 24 h (Fig. 3c, d and Supplementary Fig. 8a), and their surfaces could be composed by relative hydrophilic PEG_368_-GKLVFF (or PEG_368_-GKAAGG). Following a 24 h incubation with Aβ_42_ at 37 °C, TEM images showed that Aβ_42_ incubated with M_B_ clearly contained separate morphologies of nanoparticles and nanofibers (Fig. 3e) at 24 h, which were potentially originated from M_B_ and Aβ_42_ (Fig. 3f), respectively. However, the Aβ_42_ incubated with M_3_ and M_K_ contained main species of particulate structures entangled with some fibers (Fig. 3g and Supplementary Fig. 8b), the morphology of which was totally different from free mature Aβ_42_ fibrils. Interestingly, the statistics obtained from the fiber length taken from the TEM images of Aβ_42_, and Aβ_42_/M_B_ showed similar lengths, 225 ± 109 and 191 ± 60 nm (Fig. 3h), respectively. The statistical length of Aβ_42_/M_3_ and Aβ_42_/M_K_ was decreased to 74 ± 17 and 68 ± 13 nm, respectively, suggesting that M_3_ and M_K_ could co-assemble with Aβ_42_ and inhibit the formation of free mature Aβ_42_ fibrils, probably through KLVFF recognition and binding unit. These TEM results were in accordance with the DLS measurement (Supplementary Fig. 9).

Confocal laser scanning microscopy (CLSM) was further employed to validate the specific co-assembly of Aβ_42_ and M_3_. We first labeled M_3_ nanosweeper and Aβ_42_ with Cy5 (M_3_-Cy5) (Supplementary Fig. 10) and FITC (Aβ_42_-FITC), respectively. The M_3_-Cy5 and Aβ_42_-FITC were mixed in water and co-localized well by CLSM measurement, so was M_K_-Cy5 with Aβ_42_-FITC (Supplementary Fig. 11). However, there were no obvious co-localization observed between M_B_-Cy5 and Aβ_42_-FITC, indicating that the KLVFF induced the co-assembly of M_3_/M_K_ and Aβ_42_. In order to confirm the specificity, Cy3.5-labeled bovine serum albumin (BSA), a kind of serum albumin, accoutning for 55% of blood proteins, was utilized as a competing regent. The M_3_-Cy5, Aβ_42_-FITC, and BSA-Cy3.5 were mixed together, and observed by CLSM. The results clearly displayed that the M_3_-Cy5 co-localized with Aβ_42_-FITC, but not BSA-Cy3.5, providing strong evidence for the specific co-assembly between M_3_-Cy5 and Aβ_42_-FITC (Fig. 3i,j).

### Delivery of Aβ_42_ into cells by the nanosweeper

**Fig. 4:**
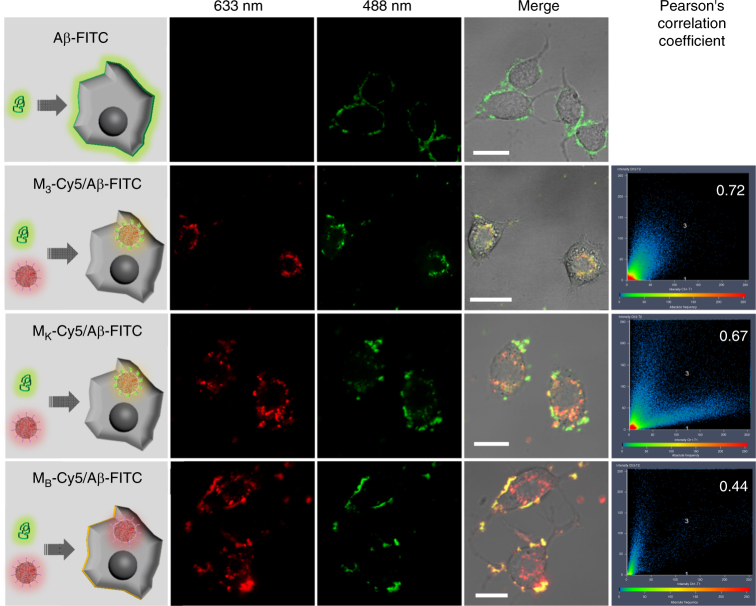
Aβ was internalized into cells with M_3_ nanosweeper. Schematic (row 1) and CLSM measurements of N2a cells treated with Aβ, a mixture of Aβ/M_3_, Aβ/M_K_ and Aβ/M_B_ (row 2, 633 nm; row 3, 488 nm; row 4, Merge). Scale bar: 20 µm

To confirm that the Aβ_42_ was substantially internalized into neutron cells by M_3_, N2a cells in a confocal petri dish (20 mm) were treated with Aβ_42_-FITC (20 μM), M_3_-Cy5, M_K_-Cy5, M_B_-Cy5 (20 μg·mL^−1^) and a mixture of Aβ_42_-FITC/M_3_-Cy5, Aβ_42_-FITC/M_K_-Cy5, Aβ_42_-FITC/M_B_-Cy5 and observed by CLSM (Fig. 4). As can be seen in Fig. 4, the Aβ_42_-FITC treated N2a cells exhibited green signal on cell surfaces, but not in the cytoplasmic matrix. This finding indicated that Aβ_42_ might get stuck in the membrane during internalization into cells, due to its hydrophobicity, which most likely led to neurotoxicity in the N2a cells. Nanosweepers, such as M_3_-Cy5, M_K_-Cy5, and M_B_-Cy5 were internalized into cells with red fluorescence in cytoplasm (Supplementary Fig. 12). As expected, N2a cells treated with Aβ_42_-FITC/M_3_-Cy5 showed green Aβ_42_-FITC fluorescence co-localized with M_3_-Cy5 (Pearson’s correlation coefficient (PCC) = 0.72) inside cells, due to the capture of Aβ_42_ by the M_3_ nanosweeper and the subsequent high-efficiency internalization into cells (Fig. 4). M_3_ increased the internalization of Aβ_42_ through co-assembly, not attaching on cell surfaces. The positive control group cells that were treated with Aβ-FITC/M_K_-Cy5 displayed the colocalized fluorescence of Aβ_42_-FITC and M_K_-Cy5 inside cells with PCC of 0.67, similar to the Aβ_42_-FITC/M_3_-Cy5 group. However, the group treated with Aβ_42_-FITC/M_B_-Cy5 (PCC = 0.44) showed red fluorescence (M_B_-Cy5) inside cells and merged Aβ_42_-FITC and M_B_-Cy5 on cell surfaces (Supplementary Fig. 13). These results suggested that M_B_ entered the cells alone, and that the Aβ_42_-FITC stuck on cell surfaces as usual. When cell surfaces were covered with Aβ_42_-FITC, the M_B_-Cy5 may no longer be able to enter cells, but stayed on cell surfaces, showing the merged fluorescence signals on cell surfaces. Therefore, the co-assembly of M_3_ and Aβ_42_ not only inhibited the Aβ_42_ fibril formation, but also increased the internalization of Aβ_42_.

### Inducement of autophagy by the nanosweeper in vitro

In order to validate the autophagy effect of M_3_ on N2a cells, autophagic structures and the marker protein LC3-II were measured using Acridine Orange staining, Bio-TEM, and western blot methods. Acridine Orange (AO) is a pH-sensitive dye and typically used as non-specific chemical agent for autophagy detection. It can mark acidic vesicular organelles with red and cytoplasm and DNA with green^30^. The ratio of red/green has been used widely to evaluate levels of autophagy^33,34^. The N2a cells were incubated with M_3_, and M_B_ and M_K_ as controls for 4 h, then stained with AO dyes for 10 min, followed by observation by CLSM. As shown in Supplementary Fig. 14a, there were many red spots in the M_3_-treated and M_B_-treated cells. In contrast, PBS and M_K_ treated cells showed few red spots. The quantification of fluorescence revealed that the red/green signal ratios of the M_3_-treated and M_B_-treated groups were remarkably higher than those of the PBS-treated and M_K_-treated cells (Supplementary Fig. 14b, ***p < 0.001).

**Fig. 5:**
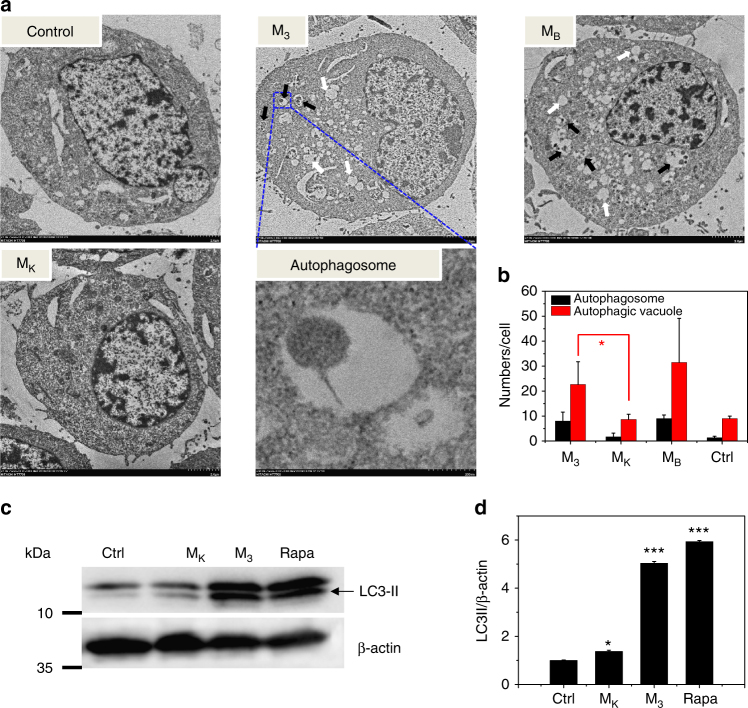
M_3_ activated autophagy significantly in vitro. **a** TEM images of N2a cells. The N2a cells were treated with PBS (control), M_K_ (200 μg/mL) and M_3_ (200 μg/mL), and M_B_ (200 μg/mL). The autophagic structures are indicated by black arrows (autophagosome), and white arrows (autophagic vacuoles). **b** The static results of autophagic structures were obtained from three random TEM images. Data are presented as mean ± s.d. (*n* = 3). **c** Western blot of LC3-II and (**d**) the corresponding quantified results. N2a cells were treated for 24 h with PBS (control), M_K_ (200 μg/mL), M_3_ (200 μg/mL), and Rapa (1 μM). Data are presented as mean ± s.d. (*n* = 3). Statistical significance is indicated as *p < 0.05, **p < 0.01, and ***p < 0.001, for comparison with control group (Student’s *t*-test)

In addition, bio-TEM, a standard method for autophagy detection, was applied to examine the autophagic structures (autophagosome and autolysosome) of N2a cells. The N2a cells were first treated with M_3_, M_K_ and M_B_ for 4 h, after which the cells were harvested and prepared for bio-TEM imaging using our previous method^35^. Finally, a JEOL JEM-1400 electron microscope was used to observe cells. As shown in Fig. 5a, an increase in the amount of small double/multi-membrane vesicles (autophagosome, black arrow) and huge vacuoles (autolysosome, white arrow) were observed in the M_3_-treated and M_B_-treated cells, compared with the PBS group. However, there were minimal autophagic structures in the M_K_-treated cells. The statistic results showed dramatic differences between M_K_ and M_3_ treated groups (Fig. 5b, *p < 0.05 Student’s *t*-test). In addition, the results of western blotting for LC3-II, an autophagy marker protein, confirmed that M_3_ increased the expression of LC3-II significantly (Fig. 5c, d). The above results lent strong support to the hypothesis that Beclin-1 residues in M_3_ could effectively induce autophagy in vitro.

### Autophagic degradation induced by the nanosweeper in vitro

**Fig. 6:**
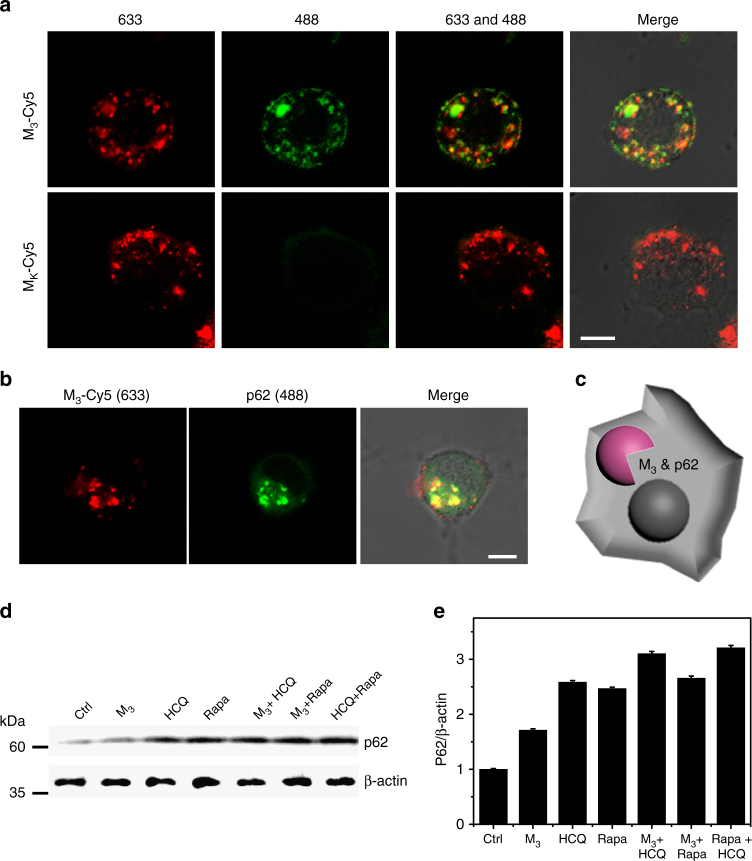
M_3_ induced autophagic degradation in vitro. **a** CLSM images showed the co-localization of the autophagosome (GFP-LC3-positive puncta, green signals) and M_3_-Cy5 (red signals). N2a/GFP-LC3 cells were treated with M_3_-Cy5 (200 μg/mL) and M_K_-Cy5 as a control for 24 h. **b** CLSM images showed the co-localization of M_3_-Cy5 (red signals) and p62 (green signals from anti-p62-labeled secondary antibody). **c** The schematic illustration of M_3_ degradation by autophagy. **d** Western blot of p62 and (**e**) the corresponding quantified results. N2a cells were cultured as follows: in regular culture medium for 24 h (lane 1); M_3_ (200 μg/mL) for 24 h (lane 2); 5 μM HCQ for 1 h (lane 3); 1 μM Rapa for 1 h (lane 4); with M_3_ (200 μg/mL) and HCQ (lane 5); with M_3_ (200 μg/mL) and Rapa (lane 6); with Rapa and HCQ (lane 7). Data are presented as mean ± s.d. (*n* = 3). Scale bar in (**a**, **b**) is 10 µm

We studied the autophagic flux of N2a cells in order to explore whether or not intracellular M_3_&Aβ_42_ adduct could be degraded by the autophagic process. M_3_ was first labeled with Cy5 (red signal). Next, the GFP-LC3 transfected N2a cells were incubated with M_3_-Cy5&Aβ_42_ (20 μg·mL^−1^/20 μM) for 4 h, followed by CLSM observation. Generally, LC3-II was recruited to the autophagosome membrane and thereby formed a green dot of GFP-LC3 when autophagy was activated^36,37^. As expected, the CLSM images revealed that most of the M_3_-Cy5 (red signal) colocalized with autophagosomes (GFP-LC3, green signal) (Fig. 6a), indicating that M_3_&Aβ_42_ was delivered to autophagosomes. Generally, the autophagosome would be fused with the lysosome, forming an autolysosome for degredation of cargo^38^. So, we therefore measured the colocalization of M_3_-Cy5&Aβ_42_ with autolysosomes. p62, An autolysosome marker protein that is preferentially degraded by autophagic process, was marked with green fluorescence through an immunofluorescence technique. The results displayed that the green immunofluorescence signals from p62 and red fluorescence signals from M_3_-Cy5 were highly merged (Fig. 6b), indicating that M_3_&Aβ_42_ was transferred into the autolysosome for degradation (Fig. 6c). To further confirm autophagic degradation, we used western blotting to detect the autophagic flux based on expression of p62. As shown in Fig. 6d, M_3_ upregulated the expression of p62, similarly to HCQ and Rapa. However, M_3_ did not induce obvious p62 upregulation of the Rapa-treated group, but increased accumulation of p62 in the HCQ-treated group, with a p62/β-actin ratio from 2.58 to 3.10 (Fig. 6e). These findings validated that M_3_ activated autophagy without blockade of the autophagic flux, indicating the possibility of autophagic degradation of Aβ_42_.

### Crossing BBB and activating autophagy in vivo

**Fig. 7:**
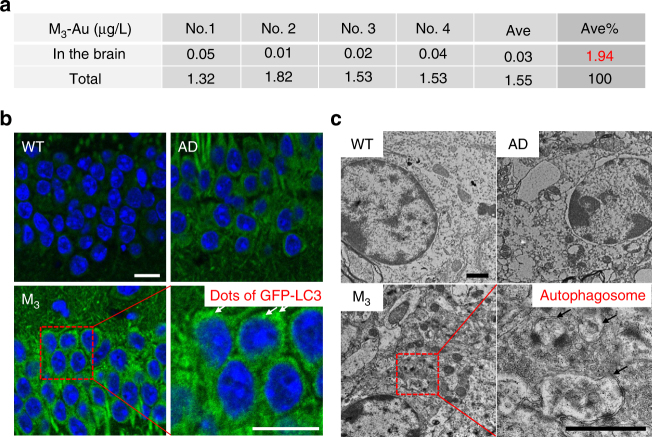
In vivo evaluation of M_3_ crossing BBB and activating autophagy. **a** The concentration of Au in the brain and in the total injection solution, respectively. The amount of M_3_ actually reached to the brain was evaluated by Au amount: M_3_ % = 0.03/1.55 × 100% = 1.94%.(*n* = 4). **b** CLSM images and **c** bio-TEM images of brain tissue slices of AD mice treated by PBS or M_3_, and WT mice treated with PBS as a control. The dots of GFP-LC3 (white arrow in **b**) and the strucuture of autophagsome (black arrow in **c**) were pointed out in M_3_ treated AD mice, respectively. Scale bar in (**b**) is 20 µm. Scale bar in (**c**) is 1 µm

For the in vivo experiment, the nanosweeper should be effectively delivered into brain. Cyclosporine was verified to effectively increase the permeability of blood-brain barrier (BBB) through influencing P-gp function in the BBB^39,40^. In order to evaluate how M_3_ nanosweeper crossed the BBB, we intravenously injected cyclosporine (10 μM) into mice, followed by i.v. administration of a dose of 200 μg·mL^−1^ M_3_ nanosweeper, which was labeled by Cy5. The mice were sacrificed and the brains were harvested for ex vivo imaging. As shown in Supplementary Fig. 15, the higher fluorenscence signal in cyclosporine and M_3_-Cy5 nanosweepers indicated that cyclosporine made more M_3_ nanosweepers reaching to the brain, compared with PBS or M_3_-Cy5 nanosweeper treated group. Furthermore, for quantifying the efficiency of M_3_ nanosweepers crossing BBB, we loaded the very small gold NPs into M_3_ nanosweepers, and i.v. injected into the mice 30 min after cyclosporine i.v. administration. The M_3_ nanosweepers distribution in the brain was characterized by gold amount, which was measured by inductively coupled plasma mass spectrometry. M_3_ nanosweepers reaching the brain could be calculated as 1.94% of total injection (Fig. 7a).

The activation of autophay in the brain determined the therapeutic effect, the immunohistochemical method was utilized for confirming the autophagic process of hippocampal neuron activated by M_3_ nanosweepers. Sections of the hippocampi from mice were stained with LC3 antibody marked with FITC. The CLSM images displayed an increased dots of green signals (autophagic structures) in the M_3_-treated mice than that of the wild type (WT) and AD groups (Fig. 7b), indicating the more LC3, the biomarker of autophagy in M_3_ treated mice brain. In addition, brain slices of AD mice treated with M_3_ were observed by bio-TEM, and the results were shown in Fig. 7c. There were a lot of autophagic structures in M_3_ treated group, but not observed in WT and AD groups without treatment. These results suggested that M_3_ could effectively induce the autophagic process in vivo.

### Clearance of Aβ and rescue of memory deficits

**Fig. 8:**
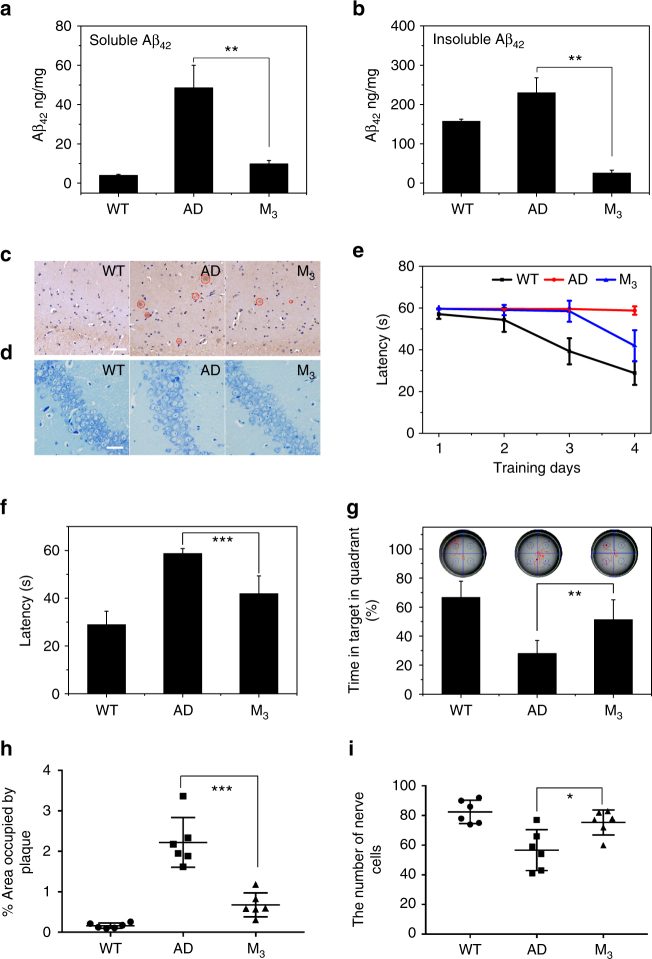
In vivo evaluation of M_3_ for the clearance of Aβ and decreasing cytotoxicity. **a** Soluble Aβ_42_ and (**b**) insoluble Aβ_42_ in the brain measured by ELISA. (*n* = 4). **c** The immunohistochemical analysis of Aβ_42_ deposition in the brains of WT control mice, AD control mice, AD mice treated with M_3_. The Aβ_42_ deposits appeared as brown signals as indicated by red dotted circle. Scale bar is 50 µm. **d** The Nissl staining of nerve cells in the brains of WT control mice, AD control mice, AD mice treated with M_3_. The Nissl bodies were stained blue. Scale bar is 50 µm. **e** The latencies of WT control mice, AD control mice, and AD mice treated with M_3_ (three mice per group). **f** The latency during the memory test in the MWM probe trial without a platform. **g** The percent (%) of time in the targeted quadrant where the platform had been located during the memory test in the MWM probe trial. **h**, **i** were the quantified results of (**c**, **d**), respectively. Data are presented as mean ± s.d. (*n* = 6). The data between AD group and M_3_ group are analyzed by a Student’s *t*-test. Statistical significance is indicated as *p < 0.05, **p < 0.01, and ***p < 0.001

In order to further study whether or not the M_3_ nanosweeper could reduce amyloid plaques in the brains of AD mice, M_3_ nanosweeper was utilized to treat APPswe/PS1dE9 transgenic mice every other day for one month^15,16,41^. Following the treatment period, the amount of soluble Aβ_42_ and insoluble Aβ_42_ in the brain tissue was measured by enzyme-linked immune sorbent assay (ELISA) (Fig. 8a,b), and confirmed that soluble Aβ_42_ and insoluble Aβ_42_ were decreased by M_3_ treatment, which was obvious lower than those of AD mice without treatment. As expected, M_3_ nanosweeper may capture Aβ_42_ to form M_3_and Aβ_42_ and enter into cells, followed by activation of autophagy and degradation of Aβ_42_ intracellluarly, leading to the decrease of soluble Aβ_42_. The decrease of soluble Aβ_42_ further resulted in the decrease of Aβ_42_ aggregation, i.e., insoluble Aβ_42._ Furthermore, immunohistochemistry analysis was carried out to label Aβ deposition in the brains. As shown in Fig. 8c, a large amount of Aβ deposition (circled with red dotted line) was observed in the AD control mice, and the difference was obvious between those and the WT control mice. Importantly, in the brains of AD mice treated with M_3_ nanosweeper, there was a significant decrease in the Aβ plaque load, indicating that M_3_ nanosweeper had the ability to reduce Aβ deposition, which was in agreement with the measurments of Aβ in the brain. The soluble and insoluble Aβ in the brain of AD mice treated with M_3_ was also decreased (Supplementary Fig. 16), but not as much as the extend of decrease in Aβ_42_, probably indicating the specificity of M_3_ for capture of Aβ_42_ by KLVFF. The results of Nissl staining of nerve cells revealed an obvious neuronal hypocellularity in the brain of AD control mice, which had small amounts of Nissl bodies (blue) (Fig. 8d). Contrastingly, M_3_ nanosweeper treatment significantly attenuated neuron loss in AD mice. Together, these results indicated that M_3_ nanosweeper could effectively decrease soluble Aβ_42_ and insoluble Aβ_42_, as well as the the deposition of Aβ and toxicity in nerve cells.

The Morris water maze (MWM) test was utilized to investigate the effect of M_3_ nanosweeper on the spatial cognitive performance of APPswe/PS1dE9 transgenic mice. The escape latencies of M_3_-treated mice for searching for the hidden platform were measured daily for 4 days. As shown in Fig. 8e, both WT mice and M_3_ nanosweeper-treated AD mice exhibited significantly shorter latency than the AD control group on days 2, 3, and 4, indicating that M_3_ nanosweeper could markedly improve the spatial memory of AD mice. When the training was completed, the place of the platform was removed and the mice were given 60 s to find the missing platform for the probe trial. As shown in Fig. 8f, the M_3_ nanosweeper-treated AD mice exhibited spatially oriented swimming behavior and shorter latencies than AD control mice. These outcomes were further confirmed by the percentage of time spent in the target quadrant (Fig. 8g). The number of times M_3_ nanosweeper-treated mice entered the small target zone was 2.4, which was greater than that of the AD mice (1.6 times). In order to evaluate long-term therapetuic effect, M_3_ nanosweeper was utilized to treat APPswe/PS1dE9 transgenic mice every other day for one month, and the MWM was tested two month post-treatment. The searching ability for the hidden platform of AD mice treated with M_3_ nanosweeper was obviously enhanced (Supplementary Fig. 17), similar as the above results, revealing that the excellent long-term therpeutic effects of M_3_ nanosweeper. The systemic toxicity of M_3_ was further investigated by hematology and histopathology assays (Supplementary Figs. 18 and 19). No significant differences were detected in pathological signs (heart, liver, spleen, lung, and kidney) between the M_3_-treated group and the control groups (WT and AD). All of above results suggested that M_3_ did not cause any obvious side effects and could be used safely as a biomaterial with excellent rescue of memory deficits in AD transgenic mice.

## Discussion

In summary, we successfully developed a multifunctional peptide-polymer based nanosweeper (M_3_), which could specifically capture Aβ via recognition and co-assembly with Aβ, followed by delivery of Aβ into cells. The nanosweeper promoted degradation of Aβ through the upregulation of autophagy. Both in vitro and in vivo experiments were performed to validate this hypothesis and the remarkable resulting anti-AD therapeutic effect. M_3_ increased the cell viability of N2a cells, which was induced by Aβ. Moreover, M_3_ decreased Aβ deposition in the brains of APPswe/PS1dE9 transgenic mice and rescued their memory deficits. The nanosweeper could have clinical practicality and provide an efficient therapeutic system for clearance of Aβ. Collectively, our findings support the potential for this new multifunctional peptide-polymer, the nanosweeper, as an promising therapeutic agent for the treatment of AD, opening up a new avenue for therapeutic applications.

## Methods

### Materials

Chitosan (CS, average Mw 5000), acryloyl chloride (TCI, Shanghai, China), Methoxy poly(ethylene glycol) (CH_3_O-PEG_n_-CH_2_CH_2_COOH) (M_n_ = 368) was purchased from Jiaxing Biomatrix and Biotechnology Inc. Thioflavine-T, Cy3.5 and Cy5 were obtained from Sigma-Aldrich. 1,1,1,3,3,3-Hexafluoro-2-propanol (HFIP) and dimethyl sulfoxide (DMSO) were purchased from Aldrich Chemical Co. and used without further purification. Aβ, FITC-Aβ, Beclin-1 (B) (>95%) and scrambled Beclin-1 peptide (B’) (>95%) were customized from GL Biochem Ltd. (Shanghai, China). Aβ_42_ was pre-treated with HFIP, followed by evaporation with N_2_ and stored at −20 °C. Cell counting kit-8 assay (CCK-8) (Beyotime Institute of Biotechnology, China) were used without further purification. Other solvents and reagents were used as received.

### Preparation of peptides

Firstly, acryl-CS was obtained as our previous reports. Peptide CFFVLKG-PEG (abbreviate to K) and CGGAAKG-PEG (abbreviate to K’) were prepared by standard solid phase peptide synthesis techniques using F_moc_-coupling chemistry. Acryl-CS (0.021 g, 0.1 mM for acrylamide bonds) was dissolved in PBS solution (2.1 mL, pH 8.0), 0.12 mM peptides in total with different ratio of m/n (1:0, 0.75:0.25, 0.5:0.5, 0.25:0.75, 0:1) were dissolved in 2.1 mL DMSO, followed by dropwise addition into acryl-CS solution under magnetic stirring. Then, the mixtures were bubbled with N_2_ for 30 min under stirring and reacted at 37 °C for 48 h in the dark. After the reaction, the resultant solution was dialyzed against deionized water (MWCO: 3500 Da) for 24 h and lyophilized to obtain a pale yellow solid. M_K_ (CS-K_0.5_-B’_0.5_) and M_B_ (CS-K’_0.5_-B_0.5_) were obtained by the same method.

### Characterization of peptides

The CFFVLKG-PEG and CGGAAG-PEG were confirmed by matrix-assisted laser desorption ionization time-of-flight mass spectrometry (MALDI-TOF-MS, Bruker Daltonics). The chemical structures of M_1–5_, M_K_, and M_B_ were proven by NMR measurements. ^1^H NMR spectra (400 MHz) of the M_1–5_, M_K_, and M_B_ in *d*^6^-H_2_O and Acryl-CS in *d*^6^-DMSO were recorded on a Bruker ARX 400 MHz spectrometer.

### ThT fluorescence assay

The ThT fluorescence assay was recorded at 485 nm through microplate absorbance reader (Tecan infinite M200, Switzerland) with excitation wavelength of 450 nm. For each measurement, the pre-treated Aβ solutions with and without M_1–5_, M_K_, and M_B_ were co-incubated with ThT solutions in phosphate buffer to the final concentration of Aβ peptide at 20 μM, M_1–5_, M_K_, and M_B_ at 20 μg·mL^−1^, ThT at 20 μM.

### Circular dichroism (CD) spectra

The CD spectra of Aβ (0.09 mg·mL^−1^, 20 μM) with or without M_3_, M_K_, and M_B_ (20 μg·mL^−1^) were monitored using a CD spectrometer (JASCO-1500, Tokyo, Japan) with a cell path length of 1 mm at room temperature. The measurements were implemented between 190 nm to 280 nm with a resolution of 1.0 nm and a scanning speed of 300 nm·minute^−1^. For each measurement, 3 spectra were collected and averaged.

### Transmission electron microscopy (TEM)

The morphologies of M_3_, M_K_, and M_B_ nanoparticles, Aβ fibril and their mixture were observed by TEM (Tecnai G2 20 S-TWIN) with an acceleration voltage of 200 kV. The M_3_, M_K_, and M_B_ nanoparticle solutions were prepared by dispersing 5 mg·mL^−1^ DMSO into PBS with a final concentration of 20 μg·mL^−1^. The Aβ nanofibril (20 μM) and the co-assembly solutions were obtained after incubating 24 h at 37 °C. 10 μL of the solutions were dropped onto a copper mesh for 5 min, subsequently removed most of the liquid through a filter paper. 10 μL of uranyl acetate solution was employed to stain the samples for 5 min, followed by drying the spare liquid with the filter. Finally, the copper mesh was washed with 10 μL of deionized water, which was blotted after staining and dried at room temperature.

### Cytotoxicity assay for mouse neuroblastoma N2a cells

Mouse neuroblastoma N2a cell line was purchased from cell culture center of Institute of Basic Medical Sciences, Chinese Academy of Medical Sciences (Beijing, China), and was utilized to evaluate the cytotoxicity of M_1–5_, M_K_, and M_B_ nanoparticles by the CCK-8 assay. A density of 5 × 10^3^ N2a cells per well were seeded in the 96-well plates in DMEM supplemented with 10% fetal bovine serum (FBS) and 1% penicillin-streptomycin in a humidified atmosphere with 5% CO_2_ and then cultured at 37 °C for a night. 10 μL of M_1–5_, M_K_, and M_B_ were dispersed in DMEM medium with a series of different concentrations, 10, 20, 50, 100, 200 μg·mL^−1^ to obtain an optimal M_1–5_, M_K_ and M_B_ concentration. Aβ at the concentration of 20 μM with and without M_1–5_, M_K_ and M_B_ sample solutions were co-incubated with cells for additional 24 h. Subsequently, 10 μL of CCK-8 solutions was added to each well and cultured for 4 h. The UV-vis absorptions of sample wells (A_sample_), A_blank_ and control wells (A_control_) were performed by a Microplate reader at a test wavelength of 450 nm and a reference wavelength of 690 nm, respectively. Cell viability (%) was equal to (A_sample_ − A_blank_)/(A_control_ − A_blank_) × 100%. All the experiments were performed in triplicate. The cell lines had been authenticated utilizing short tandem repeat DNA profiling. All cells were tested negative for cross-contamination of other human cells and mycoplasma contamination.

### Confocal laser scanning microscopy (CLSM) observation

The N2a cells incubated with and without M_3_, M_K_, and M_B_ were investigated on a Zeiss LSM710 confocal laser scanning microscope (Jena, Germany). N2a cells were seeded in complete DMEM media in a humidified atmosphere with 5% CO_2_ and then cultured at 37 °C for a night. For co-localization analysis, the medium was replaced by 1 mL of serum free fresh medium containing 20 μg (M_3_, M_K_, and M_B_) Cy5-labeled nanoparticles, 20 μM FITC-labeled Aβ. Then the cell were cultured with the serum free fresh medium for another 2 h and washed with PBS for 3 times. After replacement of the medium with PBS, cells were imaged using a Zeiss LSM710 confocal laser scanning microscope with a ×63 objective lens.

### Western blotting analysis

N2a cells were treated with M_3_, M_K_ and M_B_ (20 μg·mL^−1^) for 12 h, and then re-suspended lysis buffer solution with 1% (v/v) Triton-X 100 in 150 mM NaCl and 50 mM Tris-HCl (pH = 8.0). The protein content was estimated by a BCA kit (Applygen). Each sample (60 μg of protein) was subjected to SDS-PAGE and then transferred to a nitrocellulose membrane. Blots were blocked in a blocking buffer containing 5% (wt/v) non-fat milk, 0.1% (v/v) Tween 20 in 0.01 M TBS, and incubated with primary antibodies overnight at 4 °C and then incubated with an appropriate secondary antibody (ZSGB-BIO) for another hour at room temperature, subsequently scanned on a Typhoon Trio Variable Mode Imager. Band density was calculated using ImageJ software. The following antibodies were used: LC3B (AL221, 1:1000 dilution, Beyotime Institute of Biotechnology, China), β-actin (30101ES, 1:1000 dilution, Yeasen Biology Ltd., China) and p62 (5114, 1:1000 dilution, CST). Uncropped blots were presented in Supplementary Fig. 20.

### Bio-TEM for autophagy in vivo

The part of brain from mice were first fixed overnight at 4 °C in PBS buffer with 2.5% glutaraldehyde. After washing with PBS buffer (0.1 M) for three times, the brain tissues were fixed at room temperature with 1% osmium-containing PBS buffer for 2 h. Subsequently, all the brain tissues were washed three times with PBS buffer and dehydrated with a graded series of acetone (50, 70, 80, 90, 95, 100%) for 15 min for each step. After infiltrated with a graded of series of mixtures (acetone/EPON 812 resin: 2/1, 1/1, 1/2) at room temperature for 1 h, pure resin was added and incubated overnight at 4 °C. Finally, the gelatin capsules were used to cover the tissues and incubated with pure EPON 812 resin at 37, 45, and 60 °C for 24 h, respectively. The tissues were cut into ultrathin sections by a diamond knife and picked up with Formvar-coated copper grids (300 mesh). All of the sections were performed counter-staining with osmic acid (1%) for 1 h and uranyl acetate (4%) for 20 min, respectively. JEOL JEM-1400 electron microscope (JEOL, Tokyo, Japan) was used to observe tissues.

### Measurement of soluble and insoluble Aβ_42_ in the brain

Brain tissue (one brain hemisphere, 4 mice per group) for ELISAs was mechanically homogenized in 10 volumes of ice-cold guanidine buffer pH 8.0) and were mixed for 3 to 4 h at room temperature (RT) as described^42^. Then diluted brain homogenates with 1:10 by ice-cold casein buffer (0.25% casein/0.05%sodium azidey/20 mg/mL aprotininy/5 mM EDTA, pH 8.0/10 μg/mL leupeptin in PBS) was centrifuged 30 min at 4 °C for 16,000×*g*. The supernatant included 0.5 M guanidine in the presence of 0.1% bovine serum albumin (BSA) was removed for ELISA measurement for soluble Aβ_42_ and Aβ_total_ or stored −80 °C. The homogenate pellet that remains after centrifugation were added 425 µL cold formic acid and kept tubes on ice. Each sample was sonicated on ice continuously until that the pellet dissolved. Then performed high-speed spin at 109,000×*g* for 1 h at 4 °C and took 105 µL sample and add 1.895 mL of formic-acid neutralization buffer (1 M Tris base/0.5 M Na_2_HPO_4_/0.05% NaN_3_) on ice for ELISA measurement for insoluble Aβ_42_ and Aβ_total_ or stored −80 °C. Mouse Aβ_1–42_ ELISA Kit (R141671) and Mouse Aβ ELISA Kit (R167250) from the Trust Specialty Zeal biological trade Co., Ltd., U.S.A. were used, respectively.

### Morris water maze (MWM) experiment

Animal experiments were carried out complying with NIH guidelines for the Care and Use of Laboratory Animals, and the study the protocol was approved by the Institutional Animal Care and Use Committee of National Center for Nanoscience and Technology, China. All animals were obtained from Beijing HFK bioscience Co., Ltd. (Beijing, China). The non-Tg C57 mice were regarded as wild type mice (WT mice). The APPswe/PS1dE9 transgenic mice (AD mice) were divided into two group, AD group and M_3_ group (3 mice per group). All mice were male, and 9 month old, which intravenously administered PBS and M_3_ nanoparticles, respectively. All mice were trained and tested in a water maze with a diameter of 1.1 m. The maze was filled with water and drained daily. The temperature of the water was maintained at 22 ± 1 °C. The platform (0.1 m in diameter) was immobilized to 1 cm under the water surface during the training period, whereas the starting points were counter balanced. From the first day to the fourth day during training, the mice were measured four times from four diversed positions around the border of the maze in a semi-random order with 60 s latent period to reach the platform. If the animal find the hidden platform successfully within 60 s, it was allowed to stay on the platform for 15 s. If it failed to find the platform, the mice had to be placed on the platform for 15 s. The motional orbits of each mice was recorded and studied using a computerized video-tracking system. When the last learning trial was finished, the memory capacity of each mice were explored by a probe trial without the platform. Every trained animal was allowed to swim freely for 60 s, with two initial sites far away from the targeted platform. The time each mouse spent in searching for the platform in the quadrant where the platform used to be (target quadrant), and the number of times it crossed the target quadrant was recorded.

### Immunohistochemical analysis and nissl staining

After the MWM test, the mice were euthanized. The tissue sections in the coronal plane at 40 µm on a freezing sliding microtome from the genu of the corpus callosum through the caudal extent of the hippocampus (3 µm-thick, 4 sections/mouse, 3 mice/group). For qualitative analysis of Aβ immunoreactivity, half sections were immunostained as described^43^ with the following Aβ antibodies: Rabbit Anti-beta-Amyloid 1–42 (CT antibody) (bs-0076R, 1:200, Bioss, Beijing, China) and Anti-rabbit IgG (H + L) Aβ Hrp (074-1506, 1:200, KPL, Massachusetts, USA). The sections were observed under a microscope (XSP-C204, COIC, China). Pictures were acquired with a digital camera and analyzed with the ImageJ software. The area occupied by Aβ plaques was quantified as the percent surface area occupied in the delineated hippocampus. The plaques were quantified as the ratio of plaques vs corresponding hippocampal area and the detailed process was shown as follows: The ratio of plaques vs hippocampal area (R%) was calculated based on R% = S_t_/S × 100%. S_t_ is the total area of plaques, and the S_t_ = S_1_ + S_2_ + S_3_ + …, where the S_1_, S_2_, S_3_… indicates the area of each plaques. S is the analytical hippocampal area. For Nissl staining, half sections were stained with cresyl violet for Nissl body staining in the neurons. The sections were examined and photographed under a microscope (Nikon Eclipse E100) with DS-U3 DS Camera Control Unit. The number of staining cells was counted at 400× magnification in blinded manner, only structures of appropriate size and shape were demonstrated clearly. The final results were shown as mean ± SD (*n* = 6).

### Statistical analysis

All data are reported as mean ± standard deviation (s.d.). The in vitro experiments were performed in three independent experiments with at least three technical replicates. The in vivo experiments were performed with 3–4 mice for each group. Statistical analysis of the samples was performed using Student’s t-test, and P value of <0.05 was considered significant.

### Data availability

The data that support the findings of this study are available within the article, its Supplementary Information files and from the corresponding author upon reasonable request.

## Electronic supplementary material

Supplementary Information
