# Supplementary material for: A self-destructive nanosweeper that captures and clears amyloid β-peptides
Source: Nat Commun. 2018 May 4;9:1802. doi: 10.1038/s41467-018-04255-z (PMC5935695; doi:10.1038/s41467-018-04255-z)
Supplement: Supplementary file 1 — Supplementary Information [file 41467_2018_4255_MOESM1_ESM.pdf]

A self-destructive nanosweeper that captures and clears Amyloid β-peptides

Luo et al.

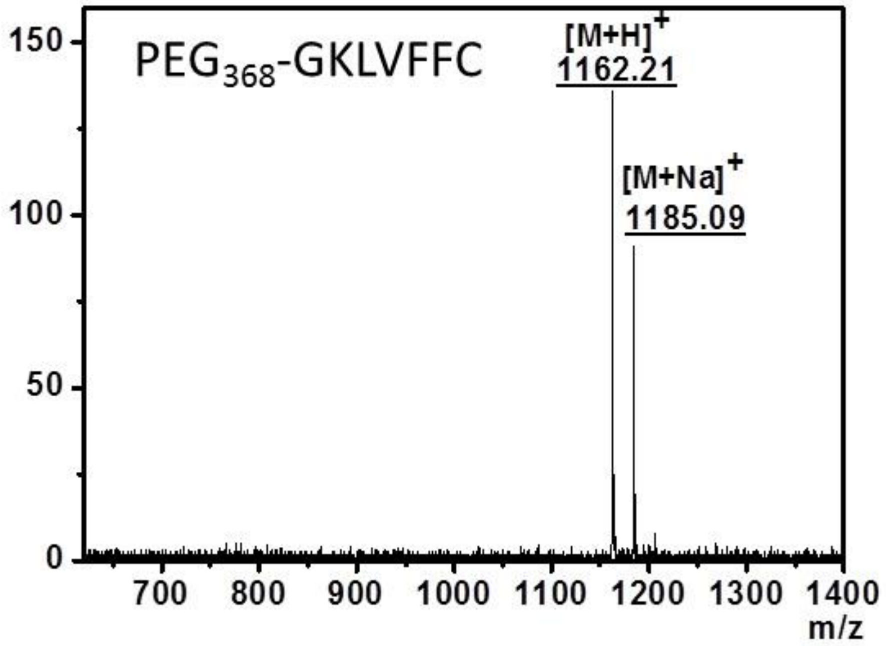

Supplementary Figure 1. The MALDI-TOF-MS spectra of PEG_{368}-GKLVFFC.

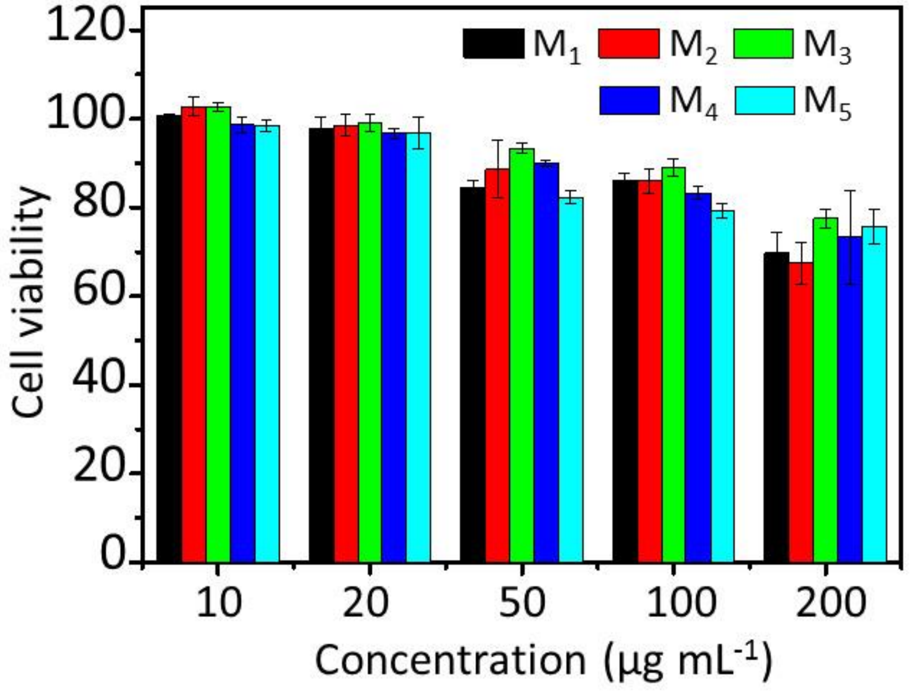

Supplementary Figure 2. Biocompatibility of $M_{1\text{-}5}$ at various concentrations by cell viability experiments. Data are presented as mean ± s.d. (n = 3).

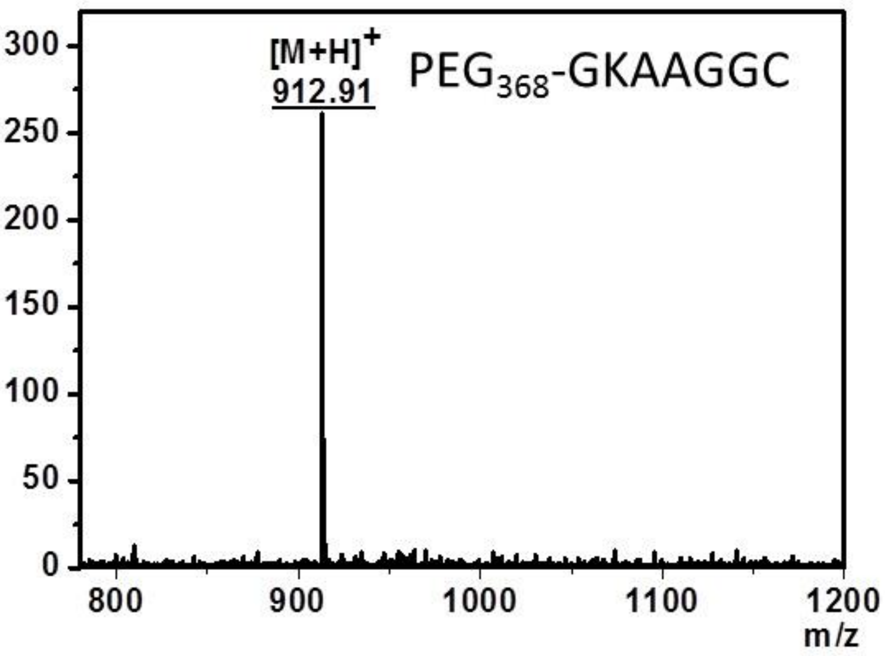

Supplementary Figure 3. The MALDI-TOF-MS spectra of PEG_{368}-GKAAGGC.

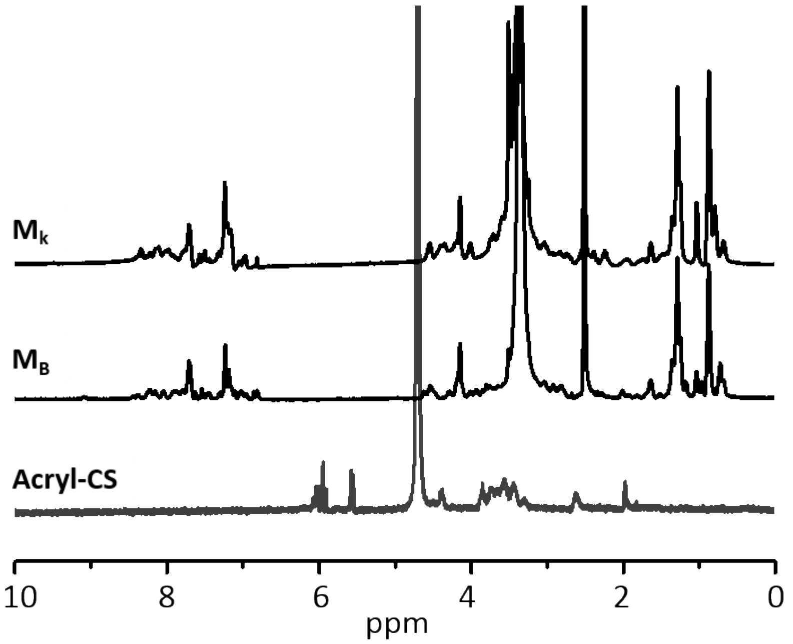

Supplementary Figure 4. ^{1}H NMR spectra of M_K, M_B and Acryl-CS.

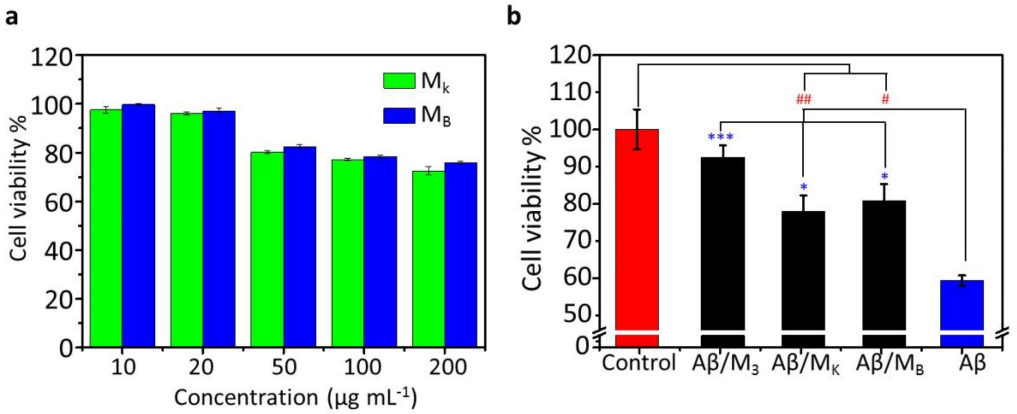

Supplementary Figure 5. (**a**) Cell viability experiments of M_K and M_B to N2a cells with different concentrations. (**b**) Reduced Aβ cytotoxicity (20 μm) to N2a cells in the presence of 20 μg·mL^{-1} M_3 , M_K (CS-$K_{0.5}$-$B'_{0.5}$) and M_B (CS-$K'_{0.5}$-$B_{0.5}$). Data are presented as mean ± s.d. (n = 3), analyzed by a Student's t-test. Statistical significance is indicated as *$p < 0.05$, **$p < 0.01$, and ***$p < 0.001$, for comparison with Aβ group, $^{\#}p < 0.05$, $^{\#\#}p < 0.01$, and $^{\#\#\#}p < 0.001$ for comparison with control group.

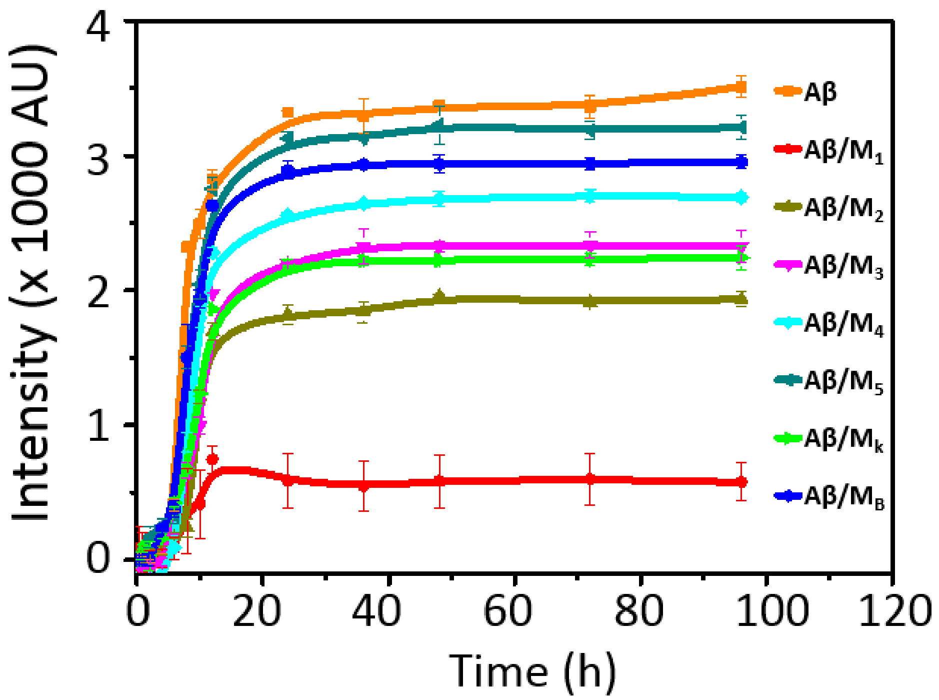

Supplementary Figure 6. The inhibitory effect by $M_{1\text{-}5}$, M_K and M_B. ThT fluorescence of $M_{1\text{-}5}$, M_K and M_B treated Aβ in 96 h. Data are presented as mean ± s.d. (n = 3).

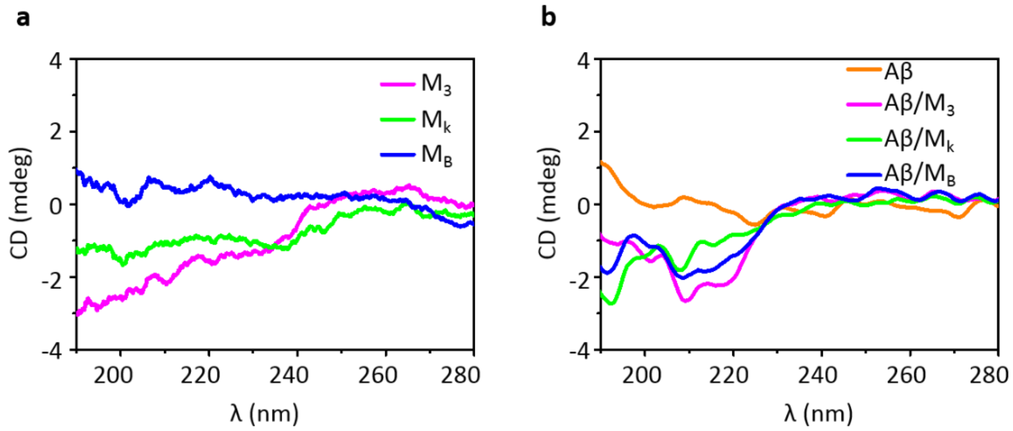

Supplementary Figure 7. (**a**) The CD spectra of M_3, M_K and M_B at 24 h. (**b**) The CD spectra of Aβ, Aβ/M_3, Aβ/M_K and Aβ/M_B at 0 h.

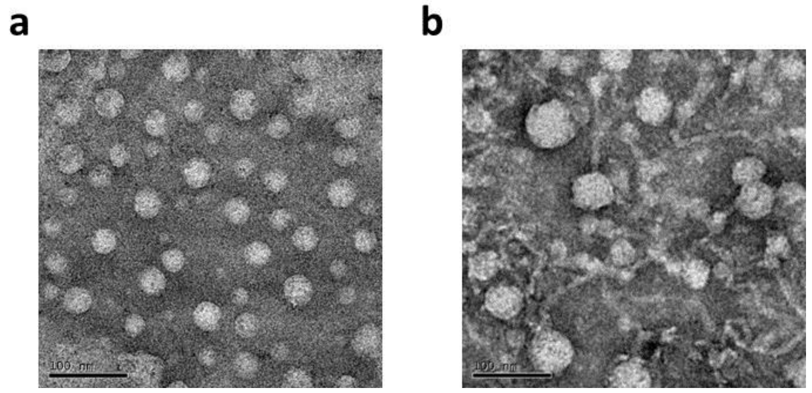

Supplementary Figure 8. TEM images of M_K (**a**) and the mixture of Aβ/M_K (**b**) at 24 h. Scale bar is 100 nm.

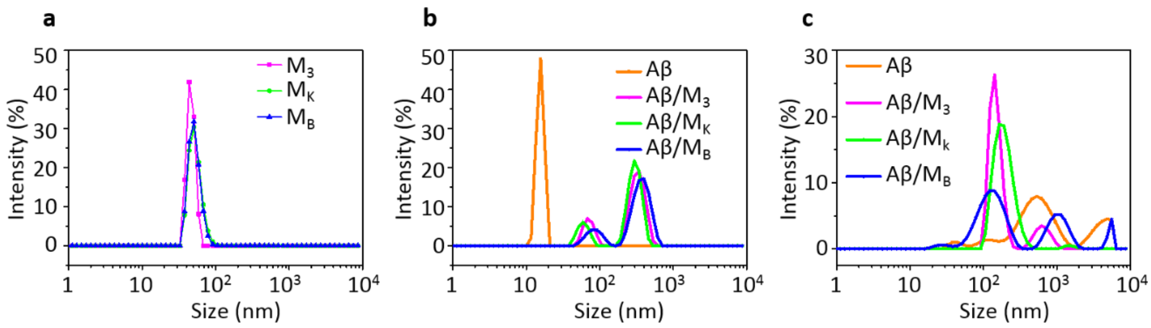

Supplementary Figure 9. (**a**) The DLS profiles of M_3, M_K and M_B at 0 h, respectively. The DLS profiles of Aβ, Aβ/M_3, Aβ/M_K, Aβ/M_B at 0 h (**b**) and at 24 h (**c**).

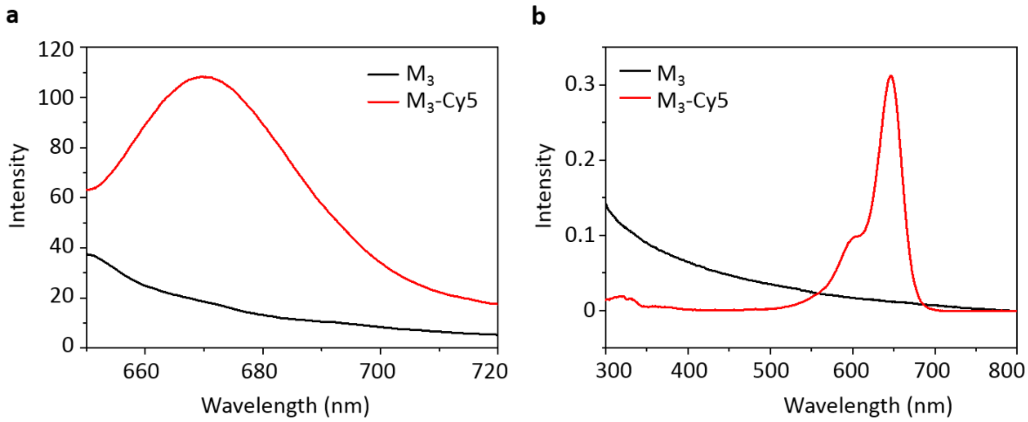

Supplementary Figure 10. (**a**) The FL spectra of M_3 and Cy5-labbed M_3 (M_3-Cy5). (**b**) The UV-vis spectra of M_3 and M_3-Cy5.

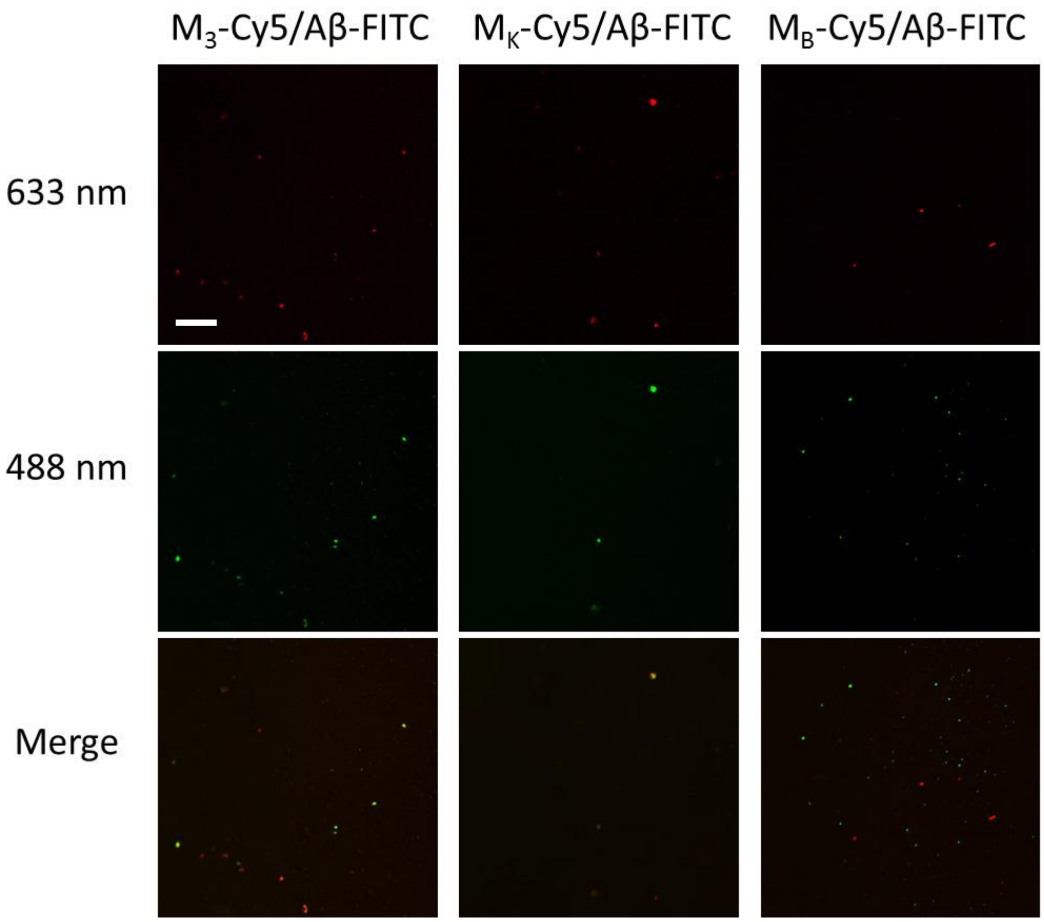

Supplementary Figure 11. CLSM measurements of Aβ-FITC treated by M_3-Cy5, M_K-Cy5 and M_B-Cy5. Scale bar is 20 μm.

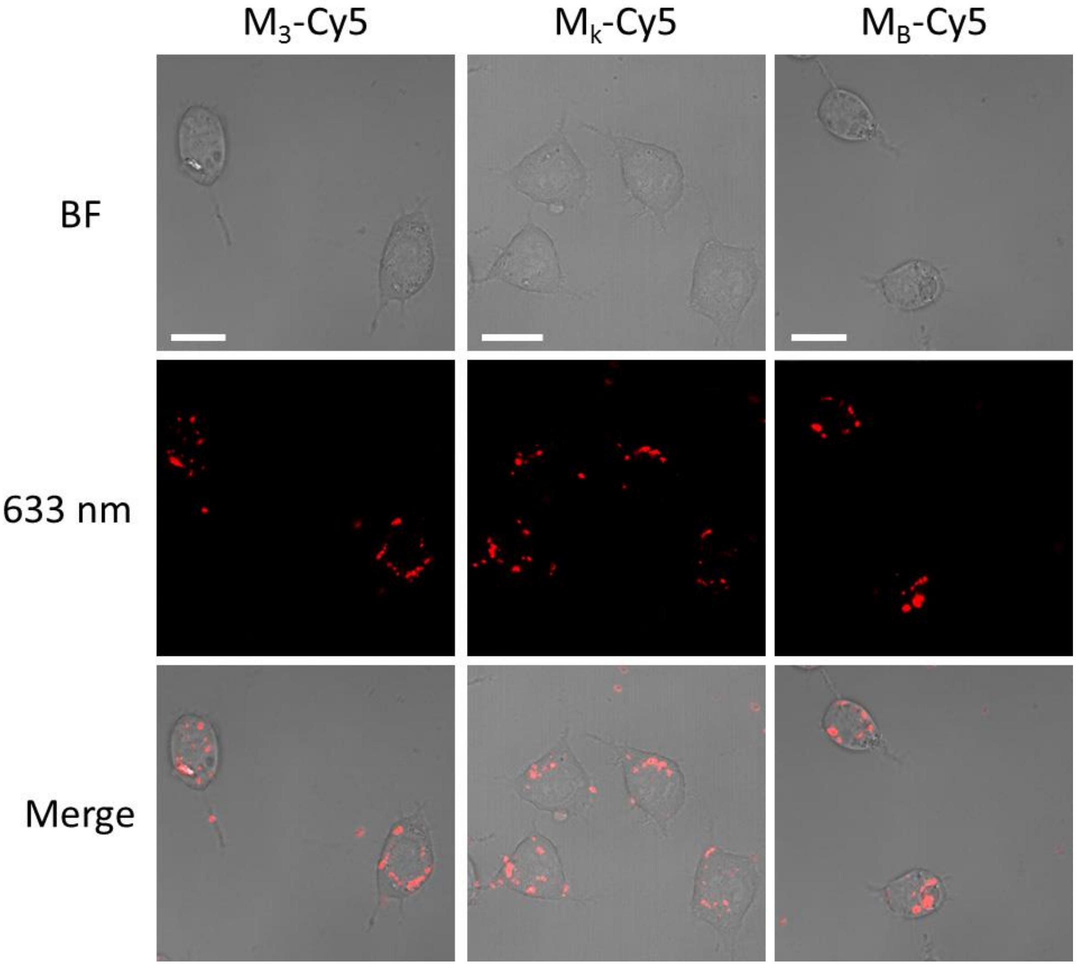

Supplementary Figure 12. CLSM measurements of N2a cells treated by M_3-Cy5, M_K-Cy5 and M_B-Cy5. Scale bar is 20 μm.

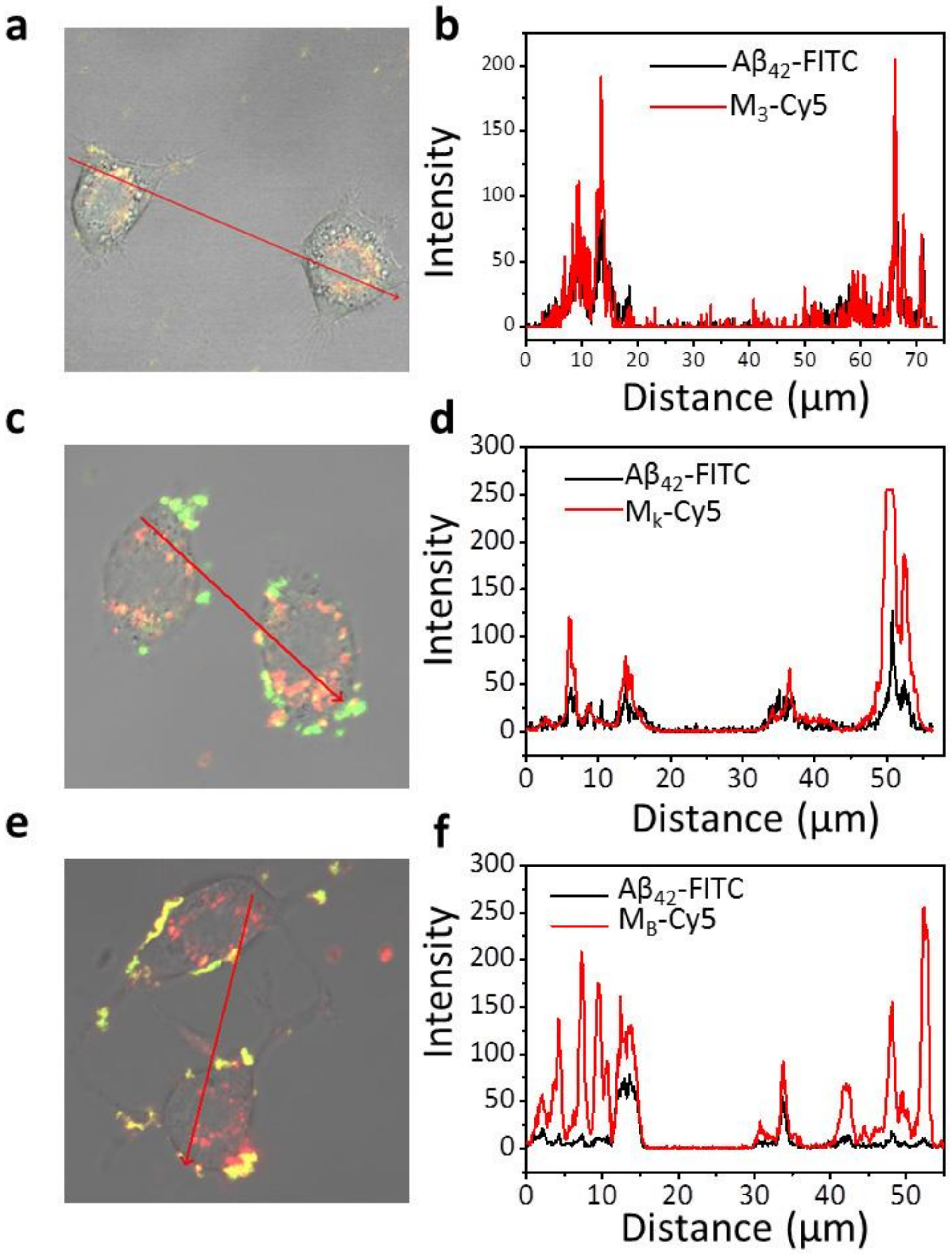

Supplementary Figure 13. CLSM measurements of N2a cells treated with a mixture of Aβ/M_3 (**a**), Aβ/M_K (**c**) and Aβ/M_B (**e**) and the normalized intensity profile of ROIs (**b**, **d**, **f**) across the red arrow in image.

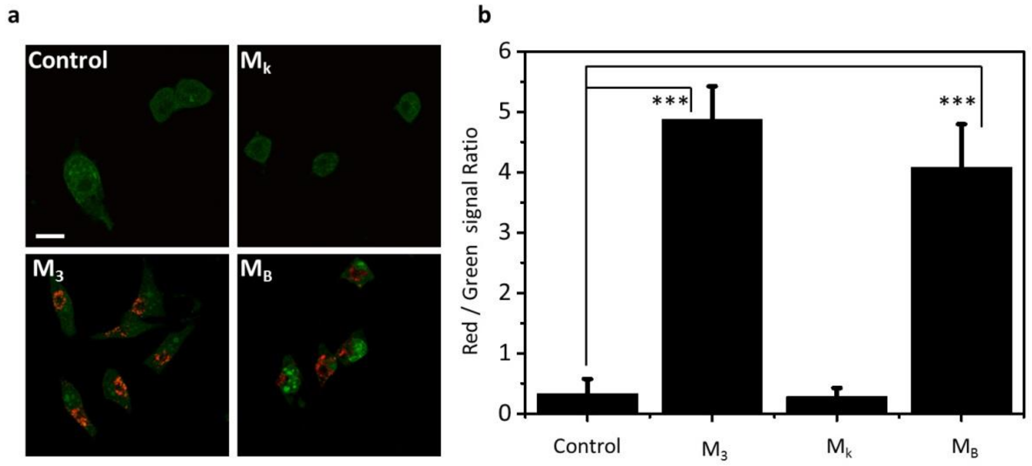

Supplementary Figure 14. (**a**) Acridine Orange (AO) staining of N2a cells. (**b**) Quantified results of red to green in (**a**). Scale bar is 20 μm. Data are presented as mean ± s.d. (n = 3), analyzed by a Student's t-test. Statistical significance is indicated as *p < 0.05, **p < 0.01, and ***p < 0.001, for comparison with control group.

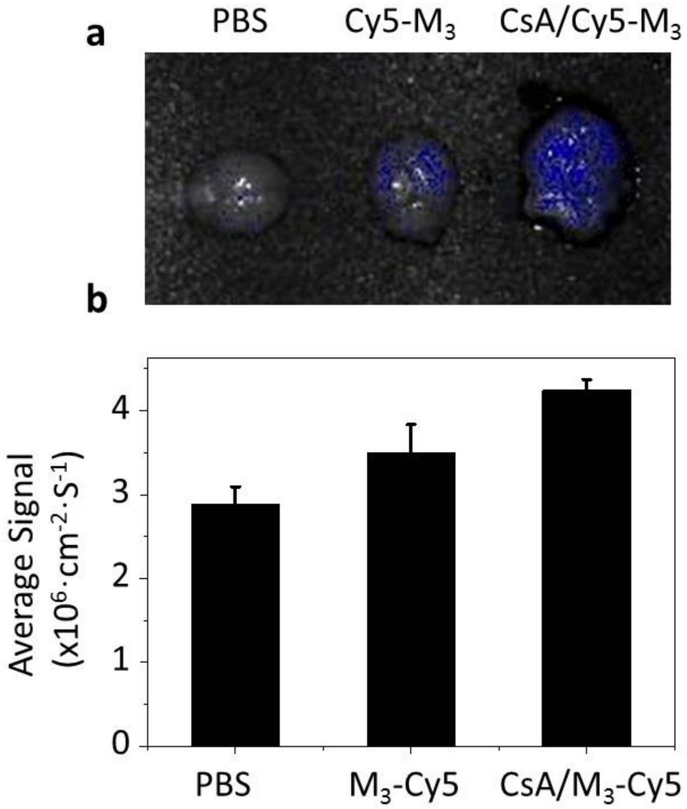

Supplementary Figure 15. (**a**) Detection of nanosweeper M_3 for crossing BBB. (**b**) Quantified results of average signal in (**a**). Data are presented as mean ± s.d. (n = 3).

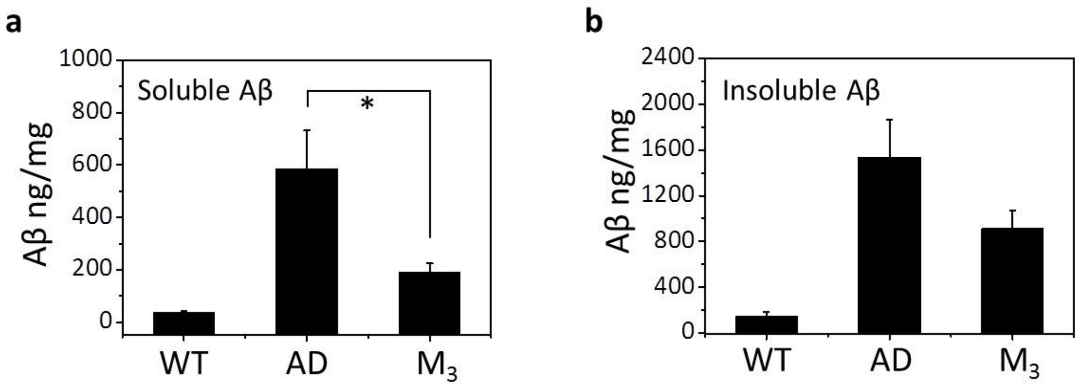

Supplementary Figure 16. *In vivo* evaluation of M_3 for the clearance of Aβ. (**a**) Soluble Aβ and (**b**) insoluble Aβ in the brain measured by ELISA. Data are presented as mean ± s.d. (n = 4). The data between AD and M_3 groups are analyzed by a Student's t-test. Statistical significance is indicated as *$p < 0.05$.

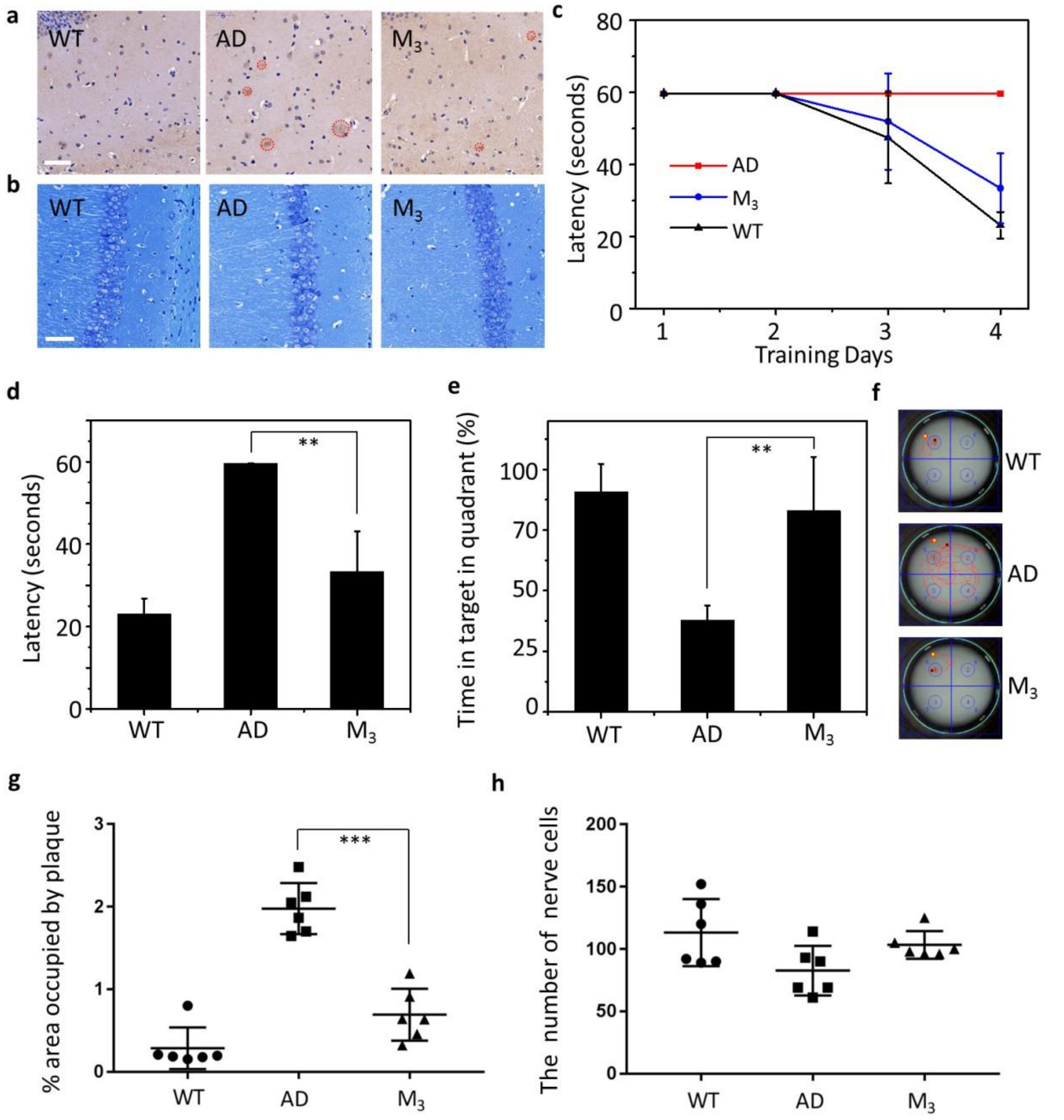

Supplementary Figure 17. *In vivo* long-term (2 month) evaluation of M_3 for the clearance of Aβ and decreasing cytotoxicity. WT control mice, AD control mice, AD mice treated with M_3 were tested 2 month post-treatment, four mice per group. (**a**) The immunohistochemical analysis of Aβ deposition in the brains of WT control mice, AD control mice, AD mice treated with M_3. The Aβ deposits appeared as brown signals as indicated by black dotted circle. Scale bar is 50 µm. (**b**) The Nissl staining of nerve cells in the brains of WT control mice, AD control mice, AD mice treated with M_3. The Nissl bodies were stained blue. Scale bar is 50 µm. (**g**) and (**h**) were the quantified results of (**a**) and (**b**), respectively. (**c**) The latencies of WT control mice, AD control mice, and AD mice treated with M_3. (**d**) The latency during the memory test in the MWM probe trial without a platform. (**e**) The percent (%) of time in the targeted quadrant where the platform had been located during the memory test in the MWM probe trial. (**f**) Typical searching trial by mice of the entire area of the pool within a limited period of time in a Morris water maze (MWM) experiment. Data are presented as mean ± s.d. (n = 6). The data between AD group and M_3 group are analyzed by a Student's t-test. Statistical significance is indicated as *$p < 0.05$, **$p < 0.01$, and ***$p < 0.001$.

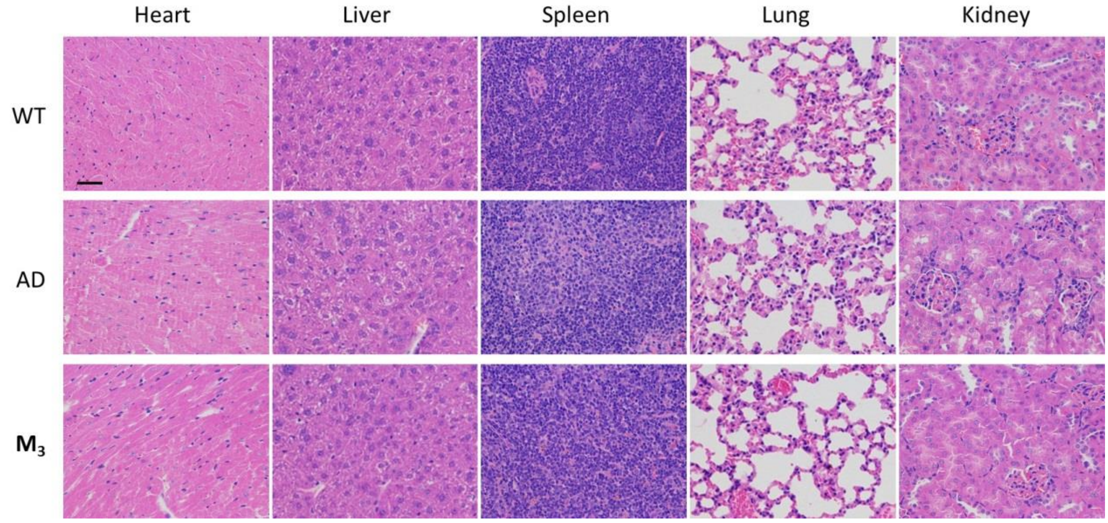

Supplementary Figure 18. Hematoxylin and eosin (H&E) images of organs of AD mice treated by PBS (AD group) or M_3 (M_3 group), and WT mice treated with PBS as a control. Scale bar is 50 µm.

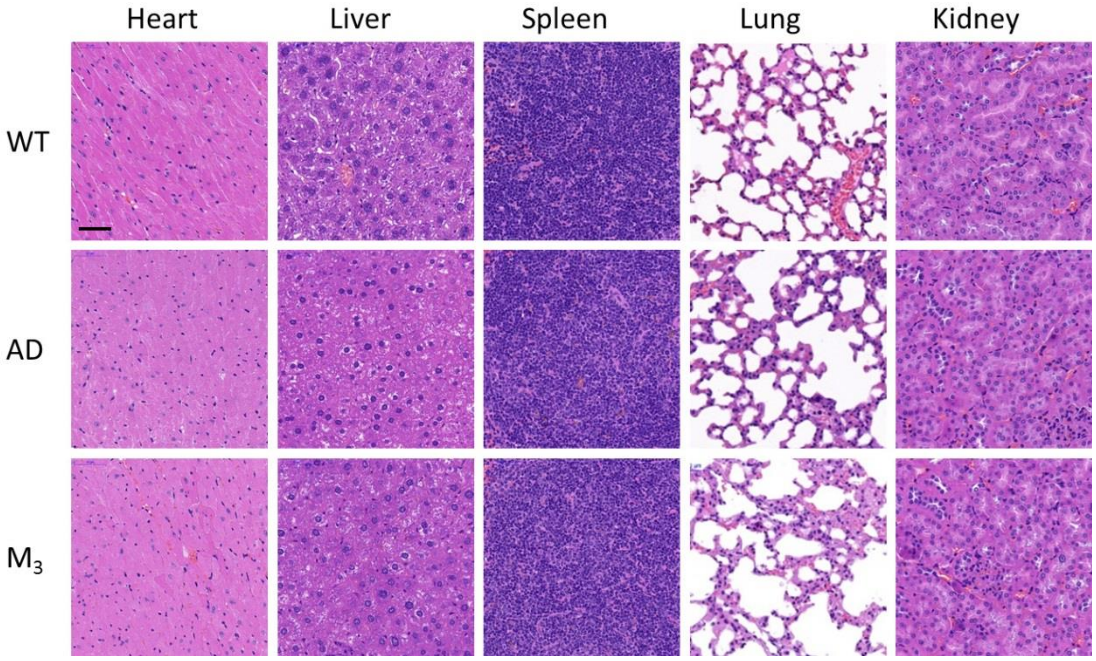

Supplementary Figure 19. WT control mice, AD control mice, AD mice treated with M_3 were tested 2 month post-treatment. Hematoxylin and eosin (H&E) images of organs of AD mice treated by PBS (AD group) or M_3 (M_3 group), and WT mice treated with PBS as a control. Scale bar is 50 µm.

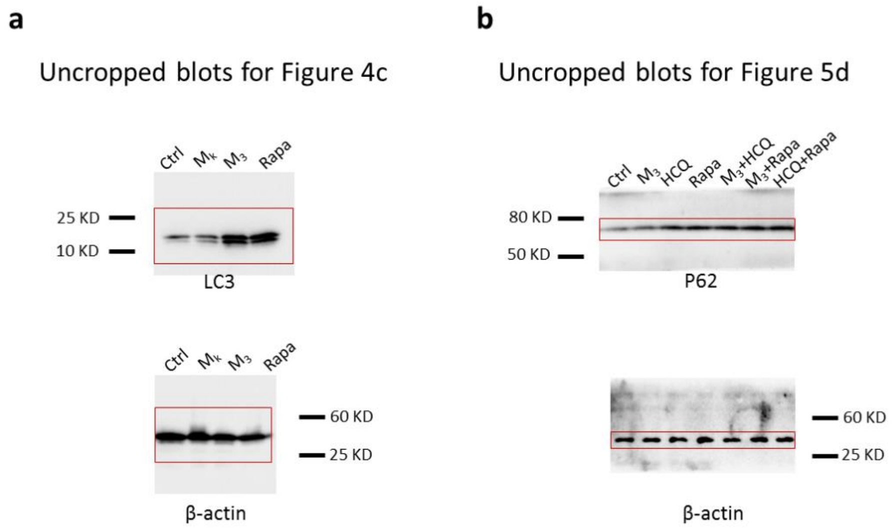

Supplementary Figure 20. Western blots (**a**) for Figure 4c and (**b**) for Figure 5e.
